# The Role of Omics Technology in Evaluating Plastic Pollution’s Effects on Plants: A Comprehensive Review

**DOI:** 10.3390/ijms262110646

**Published:** 2025-10-31

**Authors:** Irene Dini, Roberto Mancusi, Margherita-Gabriella De Biasi

**Affiliations:** 1 Department of Pharmacy, University of Naples Federico II, 80131 Naples, Italy; 2 Department of Clinical Medicine and Surgery, University of Naples Federico II, 80131 Naples, Italy

**Keywords:** microplastic, nanoplastic, metabolomics, ionomics, genomics, epigenomics, proteomics, transcriptomics, microbiomics

## Abstract

Micro and nano-plastics pose a significant threat to the global environment, affecting agricultural systems, food security, and human health. Some studies indicate that microplastics can induce physiological damage in plants, including oxidative stress, reduced germination, stunted biomass growth, and impaired photosynthesis. The extent of the damage varies depending on the type of microplastics, their size, and concentration. Moreover, micro- and nano-plastics can disturb the delicate balance of the soil microbiome. Microbial communities play a significant role in the health and functioning of ecosystems by facilitating nutrient turnover, breaking down organic matter, preserving soil integrity, and controlling diseases caused by soil-dwelling pathogens. This review highlights the role of omics technologies in elucidating the molecular mechanisms underlying plant responses to micro- and nanoplastics. The findings can enhance our comprehension of how micro- and nanoplastics affect agricultural systems when they contaminate soil.

## 1. Introduction

**Figure 1:**
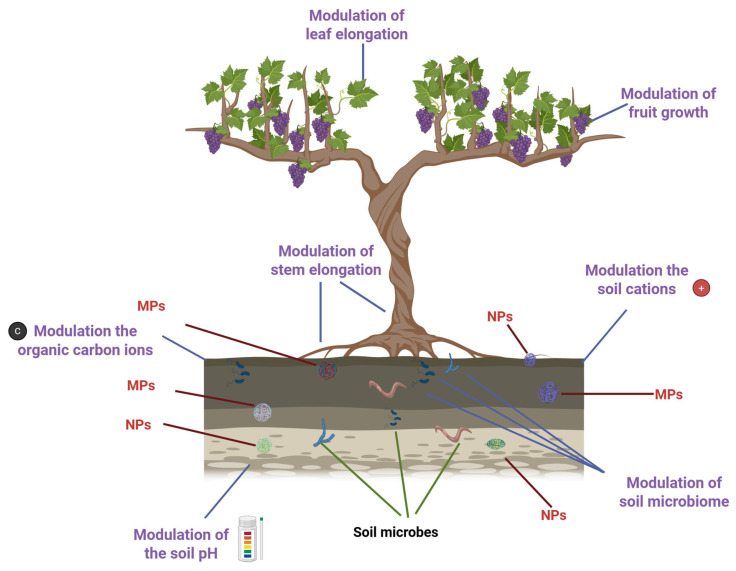
Impact of plastic derivatives on plant growth and soil properties. Created in BioRender. https://BioRender.com/wja217p (accessed on 13 October 2025).

With over 10,000 variants in use, synthetic and natural polymers have become ubiquitous in industrialized societies, underpinning the production of everything from textiles to packaging [1]. However, this convenience comes at a steep environmental cost. By 2040, global plastic waste is projected to soar to a staggering 700 million tons [2]. Despite the scale of production, plastic waste management remains alarmingly inefficient. Only about 9% of plastic waste is recycled worldwide. Roughly half ends up in landfills, and another 20% is mismanaged. Regional disparities are stark: Europe relies heavily on incineration, while in the United States, nearly 75% of plastics are disposed of in landfills [3]. This imbalance between rising production and poor recycling fuels the spread of plastic derivatives, microplastics (MPs, 100 nm to 5 mm), and nanoplastics (NPs, <100 nm), along with associated chemical pollutants. These contaminants infiltrate ecosystems through water, soil, and air, and can enter the bodies of living organisms, including plants and animals. Their presence poses serious threats to biodiversity, food safety, and human health [4]. In agricultural landscapes, plastics can accumulate in soil through the use of mulch films, protective nets, sewage sludge, and compost [5]. Once embedded, MPs and NPs can alter the speciation of heavy metals, reduce their bioavailability, and disrupt key soil parameters such as pH, cation exchange capacity, and dissolved organic carbon levels. Factors such as particle size, concentration, exposure time, and existing soil conditions (e.g., heavy metal contamination and pH) influence how plastics interact with these elements [6]. Moreover, MPs and NPs can physically block plant tissues, hinder nutrient uptake, and act as carriers for toxic additives [7], including carcinogens [8] and endocrine-disrupting compounds [9]. They can absorb organic pollutants via diffusion and partitioning, bind with heavy metals, and transport harmful microbes and antibiotic-resistant bacteria [10]. These interactions pose serious risks to both soil-dwelling organisms and human health. Over time, MPs undergo environmental aging, undergoing physical, chemical, and biological changes [11]. Their surfaces may become more hydrophilic, acquire oxygen-containing functional groups, and change their charge properties, altering their behavior and interactions with contaminants [12,13] (Figure 1).

Given their widespread presence and potential for harm, plastic waste derivatives are now recognized as emerging contaminants of concern [14]. Despite increasing attention, no standardized protocol exists for field sampling of plastic derivatives. Most studies focus on laboratory procedures, often overlooking field collection. Inadequate sampling and poor post-collection handling can lead to contamination, data loss, or misrepresentation of plastic derivatives levels. To obtain representative soil profiles, random sampling designs, plot-wise sampling (~15 g per point) [15], or composite sampling can be used. Sampling depth must reflect site-specific objectives: Topsoil (0–30 cm) is collected with stainless steel shovels or augers, while deeper layers (>100 cm) are collected with core drills. Sample size varies by analytical method; micro-FTIR may require 15 g [15], whereas µRaman requires only 0.5 g [16]. MPls lower than 4 µm (or 10 µm in reality). µRaman and nanoRaman. To prevent contamination, samples should be stored in non-plastic containers such as aluminum bags [15]. Detection of plastic derivatives typically involves two steps: cleanup to eliminate matrix interferences and characterization by spectroscopic techniques (e.g., FTIR, Raman). Cleanup methods are used to preserve the integrity of plastic derivatives and remove contaminants. They include chemical (alkaline, acidic, oxidative), physical (e.g., pressurized fluid, magnetic separation), and enzymatic approaches, and the use of a blank filter to estimate the contamination is advisable [17]. To avoid polymer degradation, physical and enzymatic methods are generally preferred. The absence of standardized methods and units complicates safety assessments and limits accurate evaluation of the distribution and impact [18]. Advances in omics technologies, such as genomics, epigenetics, transcriptomics, metabolomics, proteomics, and ionomics, are shedding light on how these pollutants affect plant health, disrupt cellular signaling, and influence broader ecological dynamics [19].

This review examines the contributions of omics technologies to our understanding of the interactions between plastic derivatives, the soil microbiome, and plants, as well as the cascading risks posed by plastic-derived pollutants across the lithosphere.

## 2. Omics Technologies

**Figure 2:**
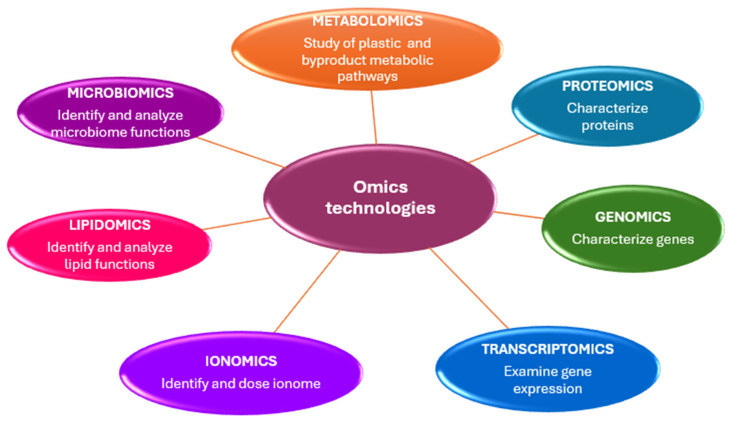
Omics technologies skills in microplastic studies.

Genomics, epigenetics, transcriptomics, metabolomics, proteomics, and ionomics are disciplines known as “omics” technologies, characterized by the suffix -omics (Figure 2).

The primary objective of omics sciences is to identify, characterize, and quantify all biological molecules that contribute to the structure, function, and behavior of cells, tissues, or organisms [20].

### 2.1. Metagenomics

Metagenomic methodologies enable the comprehensive characterization of microbial communities from diverse environmental matrices through whole-genome sequencing of total extracted DNA [21]. Microorganisms in the plant microbiome form a dynamic food web. They utilize plant-derived nutrients and contribute to nutrient cycling, detoxification, and stress resistance [22]. Through reciprocal signaling via root exudates, microbial communities engage in dynamic communication with their host, enhancing plant defenses against abiotic and biotic stressors. This intimate association has led to the conceptualization of the plant microbiome as a “second genome,” underscoring its essential role in plant health, development, and resilience [23].

#### 2.1.1. The Technique in Metagenomic Analyses

**Figure 3:**
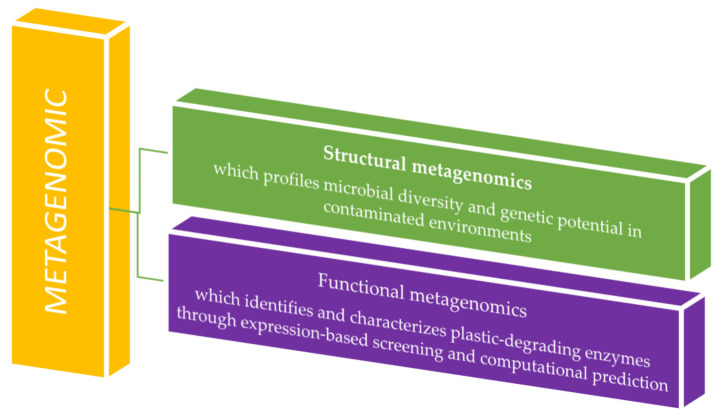
Metagenomic analysis application in plastic waste residue studies.

Metagenomics comprises structural and functional branches that together reveal microbial diversity and activity. Structural metagenomics characterizes community composition through sequencing, while functional metagenomics identifies gene functions via expression screening or computational prediction. Their integration links taxonomy to ecological roles (Figure 3) [24].

Structural metagenomics employs techniques such as PCR amplification and hybridization with degenerate primers to sequence environmental DNA, facilitating culture-free taxonomic profiling, novel gene discovery, functional prediction, and elucidation of microbial ecological roles [25]. Functional metagenomics links gene expression to phenotypic traits. Following DNA extraction, candidate genes are identified through phenotypic screening and induced expression, and then validated using metagenomic library comparison and heterologous expression systems [26]. Bioinformatic tools play a crucial role in interpreting metagenomic data. Many rely on sequence homology, aligning reads to annotated databases using exact matches (e.g., k-mers) or traditional algorithms such as BLAST (https://blast.ncbi.nlm.nih.gov/Blast.cgi, accessed on 13 October 2025) and BLAT (https://www.genomeblat.com/). Platforms like MG-RAST (https://www.mg-rast.org/) and MEGAN 5 (https://evomics.org/resources/software/metagenomics/megan/, accessed on 13 October 2025) streamline this process by aligning sequences to reference databases. MG-RAST utilizes FragGeneScan to predict open reading frames (ORFs) and aligns translated sequences to the M5NR database via BLAT. At the same time, MEGAN processes outputs from tools such as blastx/blastp, DIAMOND, or RAPSearch2 against the NR database, generating detailed taxonomic and functional profiles [27].

#### 2.1.2. Metagenomic Studies on Soil Microbial Responses to Plastic Derivatives

Recent metagenomic investigations have revealed that MPs significantly alter soil microbial diversity and abundance, impacting both bacterial and fungal communities [28]. These effects are strongly influenced by MP type, concentration, and physicochemical properties, which modulate microbial interactions and ecological dynamics [29]. MPs act as nucleation sites for biofilm formation, giving rise to the “plastisphere”, a complex, microbe-rich niche with distinct ecological and functional traits. Biofilm development and turnover reshape MP behavior and contribute to their ecotoxicological footprint [30]. Key research challenges include identifying plastisphere-specific taxa, deciphering biofilm assembly mechanisms, and understanding microbe–MP–environment interactions [30]. Comparative metagenomics has uncovered diverse microbial consortia capable of plastic degradation [31]. Notably, PET-degrading microbes employ α/β-hydrolase enzymes (e.g., PETase, MHETase, cutinase, lipase, carboxylesterase) to depolymerize PET into monomers, which are further metabolized into CO_2_, H_2_O, CH_4_, and N_2_ [32,33]. Marine strains such as *Pseudomonas aestusnigri* (via PE-H) [34] and *Ideonella sakaiensis* 201-F6 (via PETase/MHETase) [35] demonstrate efficient PET breakdown at moderate temperatures. Other taxa, including *Alcanivorax borkumensis*, *Alteromonadaceae*, and *Burkholderiales*, contribute to the degradation of LDPE and PHBH [36].

Metagenomic analyses by Sun et al. have demonstrated that polystyrene and polylactic acid MPs (diameter 100; size 154 μm), especially when co-exposed with cadmium (Cd), alter soil microbial communities. Cd amplifies shifts in dominant fungal and viral populations, while nitrogen-transforming and pathogenic genera emerge as bioindicators. High-dose PLA (size 10% *w*/*w*) enhances nitrogen metabolism, pathogenic gene abundance, and copiotrophic *Proteobacteria*, though overall MP–Cd synergism remains limited [29]. Further investigations into MPs (polyethylene, polystyrene, PLA (diameter 100; size 154 μm), and zinc oxide nanoparticles (nZnO) reveal domain-specific sensitivities. *Archaea*, fungi, and viruses respond more strongly than bacteria. High pollutant concentrations suppress genes involved in carbon and nitrogen cycling, while promoting CO_2_ fixation and sulfur metabolism. nZnO disrupts microbial network connectivity, but MPs do so particularly at elevated doses, partially buffering these structural impacts. PLA at 10% *w*/*w* induces the most severe fungal disruption, impairing nutrient-cycling functions [37]. Studies on sweet sorghum show that MPs (diameter 6.5 µm; 0.1%; size 0.5% *w*/*w*) combined with heavy metals negatively affect rhizosphere taxa involved in nitrogen (*Acidobacteria*, *Actinobacteria*) and phosphorus cycling (*Acidobacteria*) [38]. Mulch film degradation enhances microbial colonization by increasing surface adsorption and the abundance of functional groups [39]. Zhang et al. have found MP surfaces enriched with polyethylene-degrading bacteria (*Bacteroidetes*, *Actinobacteria*, *Proteobacteria*), alongside *Chloroflexi*, *Acidobacteria*, and *Gemmatimonadetes* [40]. Li et al. have reported that high-density polyethylene MPs are more detrimental than polystyrene or PLA, promoting nitrogen-cycling bacteria and eukaryotic pathogens. At the same time, their inhibitory effect on *Pennisetum alopecuroides* diminishes over time [41]. Jiang et al. have shown that high-density polyethylene, polystyrene, and polylactic acid MPs (diameter 100 μm; size 2% *w*/*w*) influence the size of barley root and rhizosphere microbiomes: nanoparticles reduce fungal diversity, while MPs increase root endophytic bacterial richness. Sensitivity varies by plant developmental stage and plastic type [42]. Huang et al. have observed that polyethylene MPs do not significantly alter overall microbial diversity, though MP surface communities exhibit lower diversity indices [43]. MPs preferentially harbor pathogenic bacteria (e.g., Pseudomonas, *Bacillus*), whose structural resilience facilitates stress tolerance and ecological risk [44]. Low-density polyethylene fragments (size 0.5% w/soil) increase nitrogen-cycling taxa, reduce endophytic associations, and enhance rhizosphere pathogenicity. Scanning electron microscopy studies have identified *Bacillus*, *Microvirga*, *Aspergillus*, and *Trichoderma* as primary colonizers. Alterations in the microbial community and the functions of the soil and rhizosphere lead to detrimental effects on plant fitness (lower chlorophyll content and higher levels of oxidative stress enzymes) [45]. Poly(butylene adipate-co-terephthalate) (diameter 56.672 µm; size 20 g kg^−1^) MPs are degraded by *Flavobacterium*, *Variovorax*, and *Microbacterium* [46]. Microbial shifts influence plant metabolism and interactions. PICRUSt-based predictions indicate elevated amino acid metabolism, xenobiotic degradation, and biofilm-mediated transport of pathogens in MP-contaminated soils [47]. Pathogen prevalence on MPs varies by polymer type, exposure duration, and location [48]. In the North Atlantic, PET plastics harbor elevated levels of *Pseudomonas* [49], *Enterococcus* abundance increases near urbanized Adriatic coastal zones, while *Escherichia* is prevalent in the Mediterranean [50]. Finally, Sun et al. have revealed that MPs facilitate horizontal gene transfer between bacteria and phages, promote taxa such as *Halobacteriaceae* and *Pseudoalteromonadaceae*, and buffer microbial communities against environmental perturbations [51].

### 2.2. Epigenomics

Epigenetics, first introduced by Waddington in 1947, initially referred to the influence of genes and their products on an organism’s traits. Today, it studies the modulation of gene expression without altering the DNA sequence [52]. These modifications have a significant impact on plant gene expression, affecting development and phenotypic plasticity [53]. Key mechanisms include DNA methylation, histone modifications, and non-coding RNAs (nRNAs) [54]. Recent studies indicate that genetically identical plants can show different DNA methylation (DM) in response to stress. Methylation patterns are influenced by stress type, genotype, tissue type, and the organism [55]. DM predominantly occurs at cytosine residues within specific sequence contexts—CG, CHG, and CHH (where H represents A, C, or T)—and plays a pivotal role in regulating gene expression by modulating transcription factor binding and chromatin accessibility [56]. As a key component of epigenetic regulation, DM, along with histone modifications and small RNAs, affects plant growth, reproduction, and stress adaptation. Manipulating these mechanisms offers promising avenues for advancing sustainable agriculture [57].

Long-term epigenetic modifications facilitate evolution and phenotypic plasticity. At the same time, short-term mechanisms enable the selection of offspring that are better suited to changing environments and able to survive under stress [58].

MPs can interfere with chromosomes, disrupting their segregation and altering chromatin structure, often leading to aneuploidy and nuclear abnormalities. They can trigger genes linked to oxidative stress and inflammation, though their effect on antioxidant enzyme genes remains inconsistent. Furthermore, MPs can influence a wide range of genes that govern essential biological processes across various cell types [59]. Yu et al. have reported that exposure to micro-/nanoplastics can reduce the expression of epigenesis-related genes in *Caenorhabditis elegans*, suggesting potential multi- and trans-generational reproductive issues linked to germline toxicity and epigenetic regulation [60].

Dainelli et al. have examined how polyethylene terephthalate NPs affect DNA methylation in *Spirodela polyrhiza*, using the methylation-sensitive amplification polymorphism technique with MspI and HpaII enzymes to target 5′CCGG sites. The study revealed increased hypermethylation, indicating a genomic defense response that may shield against reactive oxygen species or trigger new epialleles that alter gene regulation under stress [61]. Maity et al. have demonstrated that exposure of *Allium cepa* roots to nanopolystyrene results in a dose-dependent reduction in *CDC2* gene expression, thereby impairing the plant’s ability to undergo cell division [62]. Zhuang et al. [63] have investigated the impact of polystyrene microplastics (PS-MPs) and nanoplastics (PS-NPs) on *Cucumis sativus*. They have shown that PS-MPs disrupt mesophyll cell integrity, impair photosynthetic efficiency, and elevate soluble sugar levels, while altering the expression of genes involved in ATP and NADPH biosynthesis. In contrast, PS-NPs modulated *PEPC* and *PEPCK* expression, adjusting intercellular CO_2_ concentration and partially alleviating photosynthetic stress. Both particle types suppressed *NRT* and *NR* gene expression, reducing nitrogen use efficiency and inducing oxidative stress, as evidenced by increased levels of γ-GC, citrulline, and glutathione (GSH) [63].

### 2.3. Transcriptomics

Transcriptomics profiling reveals the molecular underpinnings of biological processes in both host tissues and associated microbiomes (meta-transcriptomics). It analyzes the RNA transcripts, including protein-coding mRNAs and regulatory non-coding RNAs [64]. The presence of a transcript does not guarantee functional protein translation, and the inherent instability of RNA, particularly mRNA, necessitates rapid and precise handling of the sample. To ensure data integrity, it is critical to efficiently deplete ribosomal RNA (rRNA) and remove non-target molecules that may obscure meaningful signals. Rigorous experimental design, including time-series sampling, can enhance the resolution of transcriptomic analyses [65].

#### 2.3.1. The Technique in Transcriptomic Analyses

Transcriptional profiling techniques are categorized into targeted approaches, which focus on predefined gene sets using array-based platforms, and untargeted strategies, such as RNA sequencing, which enable comprehensive analysis of the transcriptome [66].

**Figure 4:**
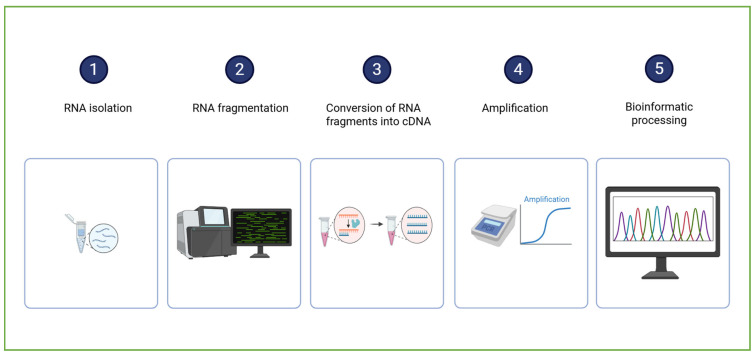
A schematic representation of RNA sequencing sample analysis and data processing. Created in BioRender. Dini, I. (2025) https://BioRender.com/0fx2v1f.

The RNA sequencing (RNA-Seq) workflow (Figure 4) begins with the isolation of total RNA from the biological sample (encompassing both messenger RNA (mRNA) and various non-coding RNA species). The RNA is then reverse-transcribed into complementary DNA (cDNA), a more stable molecule suitable for sequencing. The resulting cDNA is fragmented and ligated with sequencing adapters. The sequencing process generates millions of short reads, which are subsequently subjected to bioinformatic processing to identify the transcript origin and quantify the gene expression [67].

Recent strides in bioinformatics have ushered in powerful analytical frameworks, among which Weighted Gene Co-expression Network Analysis (WGCNA) stands out as a transformative tool for decoding complex biological traits. WGCNA leverages the principle that functionally related genes tend to exhibit coordinated expression patterns, enabling the construction of gene correlation networks that reflect underlying biological processes. By analyzing high-dimensional transcriptomic data, WGCNA identifies modules of co-expressed genes and pinpoints hub genes, central players within these modules, that may serve as key regulators or biomarkers [68].

#### 2.3.2. Transcriptomic Studies on Plant Responses to Plastic Derivatives

WGCNA studies have revealed that polyethylene mulch films negatively impact wheat physiology by suppressing photosynthesis and carbon fixation, and upregulating antioxidant enzyme activity [69].

Transcriptomic and metabolomic studies have demonstrated that polyether polyurethane mulch significantly impairs wheat growth during the seedling stage. The disruption of cellular membranes compromises key metabolic pathways in wheat roots (glycolysis and the pentose phosphate pathway), leading to altered enzyme expression, reduced ATP synthesis, and diminished energy availability. Additionally, mechanical damage alters the expression of genes involved in lignin biosynthesis and induces oxidative stress, as evidenced by elevated ROS levels. In response to this stress, wheat roots activate the jasmonate signaling pathway and increase lipoxygenase activity. In contrast, bio-based polyester polyurethane mulch shows no significant phytotoxicity. Wheat seedlings grown with this biodegradable material show enhanced tolerance to oxidative stress by elevating jasmonic acid levels, fine-tuning the antioxidant enzyme system, and stimulating γ-aminobutyric acid (GABA) metabolism. These adaptations effectively reduce ROS accumulation and support healthy growth [70].

In *Oryza sativa*, MPs stress elicit cultivar-specific transcriptional responses (genes associated with the tricarboxylic acid cycle are downregulated in *Oryza sativa* L. II You 900 hybrid indica rice plants, whereas they are upregulated in Xiushui 123 *Oryza sativa* L. hybrid japonica rice plants [71]), suggesting differential metabolic resilience. Exposure to polystyrene MPs disrupts the expression of genes linked to photosynthesis, ribosomal function, and stress signaling pathways, indicating broad physiological interference [72].

In *Nicotiana tabacum*, polyethylene MPs suppress genes involved in light harvesting and photosystem integrity; yet, specific transcripts are upregulated, suggesting an intrinsic self-protective mechanism that may aim to mitigate photodamage [73]. Similarly, Kyoto Encyclopedia of Genes and Genomes (KEGG) pathway analysis in *Torreya grandis* revealed that differentially expressed microRNAs and genes predominantly affect the photosynthesis pathway, drawing parallels to stress responses observed in algal systems [74].

In *Lactuca sativa*, studies demonstrated that charged MPs penetrate leaf tissues primarily via stomatal entry and cuticle disruption. Positively charged MPs (MP^+^) exhibit greater uptake and toxicity than their negatively charged counterparts (MP^−^), triggering a cascade of physiological, transcriptomic, and metabolomic alterations. These include reduced biomass, decline in photosynthetic pigments, elevated ROS, and enhanced antioxidant enzyme activity. Notably, MP^+^ exposure upregulates genes associated with the circadian rhythm, potentially exacerbating stress sensitivity and contributing to its heightened phytotoxicity [75].

### 2.4. Metabolomics

Metabolomics is the comprehensive study of the metabolome, the collection of small molecules (typically under 1500 Da) produced through metabolic activity. Metabolomics provides a molecular snapshot of an organism’s current state, assessing physiological changes and treatment effects by comparing control and experimental samples [76]. Metabolomic studies can be targeted or untargeted. Targeted metabolomics studies focus on specific metabolites, enabling accurate quantification. They are used to detect metabolites related to stress tolerance. Non-targeted metabolomics enables the identification of a wide range of metabolites in a single analysis. It provides less accurate dosages of individual metabolites but provides a comprehensive overview of the metabolome. Non-targeted metabolomic technology is employed to investigate the plant’s responses to abiotic stresses, including those induced by plastic-derived pollutants. Targeted metabolomics employs selective extraction protocols to quantify predefined metabolites, whereas untargeted metabolomics requires comprehensive extraction to capture the full metabolic spectrum. Both approaches utilize analytical platforms such as NMR, GC-MS, and LC-MS for data acquisition. Due to its exploratory nature, untargeted metabolomics demands more extensive data processing, including peak alignment, normalization, and compound annotation. Downstream analyses—such as bioinformatics, enrichment analysis, pathway mapping, and metabolic network reconstruction—are integral to both strategies for interpreting biological significance [77,78]. Combining targeted and non-targeted metabolomics was employed to elucidate how plastic-derived pollutants interfere with the metabolism of amino acids, sugars, organic acids, secondary metabolites, and hormones [77,78] (Table 1).

#### 2.4.1. Techniques Used in Metabolomic Analyses

**Figure 5:**
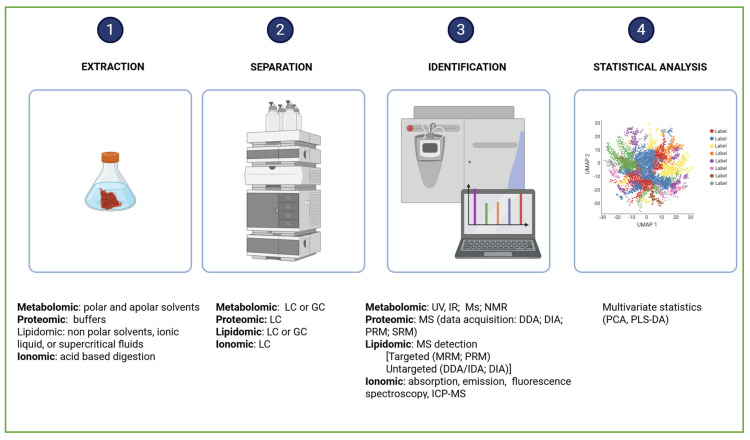
Comprehensive workflow of omics analysis. Created in BioRender. Dini, I. (2025) https://BioRender.com/h412ck2.

Metabolomics studies begin with experimental design, followed by sample preparation, data acquisition, and data analysis, culminating in compound identification and/or dosage determination. Finally, the biological interpretation reveals how organisms respond to environmental or experimental conditions (Figure 5).

Metabolomic data are generated using a combination of chromatographic methods, such as Gas Chromatography (GC) and Liquid Chromatography (LC), employed to separate the complex mixtures of metabolites, and spectroscopic techniques, including Mass Spectrometry (MS) and Nuclear Magnetic Resonance (NMR), used to identify and quantify the compounds [79,80,81,82]. The choice of chromatographic and spectroscopic techniques depends on the physicochemical properties of the metabolites [83]. Gas Chromatography–Mass Spectrometry (GC-MS) is used to profile volatile compounds. It employs trimethylsilyl (TMS) and tert-butyldimethylsilyl (TBDMS) groups to enhance the volatility and stability of metabolites, as well as ionization methods—such as electron and chemical ionization to fragment molecules for precise identification. Liquid Chromatography–Mass Spectrometry (LC-MS) is employed to analyze both polar and apolar metabolites. It provides superior reproducibility compared to GC-MS. The identification of metabolites is facilitated by comparing experimental spectra with those stored in spectral libraries, which serve as molecular fingerprints. Univariate statistical tests (e.g., *t*-tests and ANOVA) are used to assess the abundance of individual metabolites in targeted analyses. The abundance of global datasets in untargeted metabolomics is obtained by multivariate statistical tests (e.g., principal component analysis, partial least squares discriminant analysis, and orthogonal projections to latent structures discriminant analysis) [84]. Partial Least Squares Regression (PLS-R) and Partial Least Squares Discriminant Analysis (PLS-DA) are discriminant-type analyses used to categorize metabolites based on their attributes PLS. Feature selection is employed to reduce data dimensionality [83]. Over the past two decades, omics research has produced vast datasets, prompting the development of bioinformatics tools to manage this complexity. Prominent metabolic pathway databases and visualization platforms include BioCyc (https://biocyc.org/), KEGG (https://www.genome.jp/kegg/, accessed on 13 October 2025), GNPS (https://gnps-explorer.ucsd.edu/MSV000093116?dataset_accession=MSV000093116&metadata_source=DEFAULT&metadata_option=, accessed on 13 October 2025), Reactome (https://reactome.org/), and MetaCyc (https://metacyc.org/) [85].

#### 2.4.2. Plastic Derivatives’ Impact on the Modulation of Amino Acids

Amino acids play a crucial role in maintaining cellular osmotic balance and facilitating the transport of ions across membranes. They influence stomatal behavior, act as precursors for defense-related metabolites, and serve as key signaling molecules that orchestrate plant responses to environmental cues and stress conditions [86]. Exposure to MPs can severely disrupt the metabolism of critical amino acids, including glycine, threonine, serine, aspartate, alanine, and glutamate. These disturbances compromise the biosynthesis of essential proteins and metabolites, undermining plant growth, stress tolerance, and overall physiological integrity [87,88]. The metabolic disruptions triggered by microplastics do not play out the same way in every plant. Metabolomic studies reveal that MP contamination in rice significantly reduces leaf biomass, indicating impaired growth [89]. In pepper plants, polyethylene MPs reduce levels of key amino acids (about 10% of the total) involved in stress response, protein synthesis, and growth regulation, thereby compromising physiological resilience under environmental stress [90]. In *Zea mays*, MPs stimulate the release of rhizosphere amino acids and other metabolites, reflecting shifts in microbial activity and altered root exudation. These changes may disrupt plant–microbe interactions, soil fertility, and broader ecosystem dynamics [91].

#### 2.4.3. Plastic Derivatives’ Impact on the Saccharide Modulation

Saccharides play a vital role in plant defense, acting as stabilizers of cell membranes and scavengers of harmful reactive oxygen species (ROS). Metabolomic studies reveal that MPs may impair cellular energy flow by negatively regulating the glycolytic pathway [92]. Interestingly, not all interactions with micro- and nanoplastics are detrimental. In cucumber leaves, low concentrations of polystyrene NPs have been shown to enhance carbohydrate synthesis and increase the production of membrane-stabilizing metabolites [93]. In *Stevia rebaudiana*, exposure to polystyrene NP increases the levels of steviol glycosides, chlorophylls, and carotenoids [94].

#### 2.4.4. Plastic Derivatives’ Impact on the Organic Acid Modulation

Organic acids in plants drive electron and proton transfer through vital redox reactions involving NAD, NADP, glutathione, and ascorbate. Their metabolism negatively affects the cellular redox landscape, fuels the generation of reactive oxygen and nitrogen species, and influences signal transduction pathways. The organic acids also fine-tune photosynthesis and respiration. The malate acts as a key shuttle, transporting redox species from chloroplasts to the cytosol during photosynthetic activity [95]. However, exposure to MPs destabilizes redox homeostasis and impairs core physiological functions [96]. Metabolomics studies reveal that polystyrene NPs can disrupt core metabolic pathways in wheat, and that plastic derivatives upregulate threonic acid, boric acid, butanedioic acid, glycolic acid, aconitic acid, and malic acid [97]. In spinach, reduced levels of hydroxybenzoic acid compromise cell wall integrity and structural stability [98]. Cucumber responds to NP exposure by downregulating citric acid concentrations [99]. In rice, MPs downregulate picolinic acid production, weakening the plant’s resilience to external stressors [100]. The decline in organic acids in rice leaves following inoculation with MPs has been linked to impaired photosynthesis and respiration [101].

#### 2.4.5. Plastic Derivatives’ Impact on the Secondary Metabolites and Hormone Modulation

Phytohormones are regulators of plant adaptation. They affect responses to stress, maintain ionic balance, scavenge ROS, adjust osmotic pressure, and drive plant growth and development [102]. MP/NP exposure has been shown to impair the phenylpropanoid pathway, which is critical for the production of flavonoids, lignans, and other defense-related metabolites [103]. In lettuce, polystyrene MPs stimulate sphingolipid metabolism and boost the synthesis of flavonoids, ascorbic acid, and terpenoids [104]. In contrast, low-density polyethylene MPs increase anthocyanin levels in broccoli and radish sprouts, thereby enhancing their antioxidant potential [105]. In barley, polystyrene MPs downregulate the auxin production [106]. Polyester MPs upregulate hormone levels in barley and downregulate them in cucumber [107]. In rice, polystyrene MPs suppress jasmonate and lignin synthesis by modifying gene expression [108]. Soybeans respond to NP contamination with decreased levels of L-tryptophan and salicylic acid, key precursors of auxin [109]. Barley downregulates indole-3-butyric acid, indole-3-acetic acid, and dihydrozeatin, and upregulates trans-zeatin [110].

#### 2.4.6. Plastic Derivatives’ Impact on Heavy Metal Tolerance in Plants

MPs can act as carriers for heavy metals. Their surface charge, often negative due to low polarity, attracts positively charged metal cations through electrostatic interactions. This binding mechanism enables MPs to adsorb heavy metals efficiently, thereby influencing their distribution and mobility in soil. As a result, MPs can alter soil health and the dynamics of contaminants in terrestrial ecosystems [111]. Metabolomics was employed to investigate the relationships between metals, NPs, and plant metabolites. Plants are susceptible to. Elevated concentrations of heavy metals can disrupt antioxidant defenses, impair seed germination, damage cellular membranes, stunt seedling development, and cause chromosomal abnormalities that may lead to plant death. Although zinc, copper, and iron are vital for plant growth, excess can disrupt cellular function. Non-essential heavy metals such as mercury, chromium, cadmium, and lead pose even greater risks, threatening both plant health and the safety of agricultural products [112]. Metabolomic studies have revealed that the combined presence of polyethylene or polypropylene MPs (diameter 6.5 ± 2 μm, 7.8 ± 2 μm) and cadmium can negatively affect carbohydrate, amino acid, and lipid metabolic pathways, decreasing photosynthesis and activating oxidative stress responses [113]. In rice, exposure to metals and MP triggers significant shifts in leaf metabolism, with the severity of the changes closely tied to dosage levels [108]. Maize seedlings exposed to both sodium chloride and MPs (diameter 38.49  ±  0.3 nm) exhibit elevated reactive oxygen species levels and compromised photosynthetic efficiency [114].

**Table 1:** Regulation of plant metabolites in response to plastic derivative pollution.

Plant	Plastic Derivatives	Plastic Derivatives Levels	Effect	Reference
* Capsicum annuum *	polyethylene terephthalate (150 µm)	20 mg · kg^−1^ and 200 mg · kg^−1^	down-regulation levels of amino acids	[90]
* Zea mays *	polystyrene (100 nm) and polypropylene (10 µm)	2% of soil mass	up-regulation of amino acids	[91]
* Solidago canadensis *	mixture of polyethylene pellets, fragments, and fibers (0.60 mm–1.00 mm)	0.5% of the soil weight	down-regulation of carbohydrates	[92]
Cucumber	polystyrene (50–100 nm)	50 and 100 mg/L	down-regulation of carbohydrates	[93]
* Stevia * *rebaudiana*	polystyrene (20 nm)	10 mg/L	up-regulation of steviol glycosides	[94]
* Triticum aestivum *	polystyrene (120–254.4 nm)	0.1 mg/L	Upregulation of threonic acid, boric acid, butanedioic acid, glycolic acid, aconitic acid, malic acid	[97]
* Spinacia oleracea *	CeO_2_ NPs (194.8–215.3 nm)	0.3, and 3 mg/plant.	downregulation of 3-hydroxybenzoic acid, 4-hydroxybenzoic acid, and nicotinic acid	[98]
* Cucumis sativus *	nano-Cu (2590 nm)	10 and 20 mg/L	downregulation of citric acid	[99]
* Oryza sativa *	polybrominated diphenyl ethers	500 μg/L	down-regulation of amino acids	[89]
* Oryza sativa *	Di-(2-ethylhexyl) phthalate	80, 160, and 320 ng/mL	downregulation of picolinic acid	[100]
* Oryza sativa *	polystyrene (<50 µm)	50 mg L^−1^ and 250 mg L^−1^	downregulation of organic acid	[101]
* Oryza sativa *	polystyrene (~20 nm)	10, 50, and 100 mg L^−1^	downregulation of jasmonate and lignin	[108]
* Lactuca sativa *	polystyrene (0.2 µm)	10, 20, 30, 40, and 50 mg L^−1^	upregulation of flavonoids, ascorbic acid, and terpenoids	[103]
Broccoli	low-density polyethylene (<2000 μm)	0.01 mg L^−1^–10,000 mg L^−1^	downregulation of the glucosinolates; upregulation of anthocyanins	[104]
Barley	polystyrene (<5 mm)	2 g mL^−1^	downregulation of auxins	[105]
Barley and cucumber	polystyrene (790 nm–4999 nm)	0, 100, and 1000 mg L^−1^	up-regulation of hormones in barley and down-regulation in cucumber	[106]
Barley	polystyrene (<5 mm)	2 g mL^−1^	Downregulation of indole-3-acetic acid, indole-3-butyric acid and dihydrozeatin	[110]
*Glycine max*	polystyrene (~73 nm)	75 mg L^−1^	downregulation of salicylic acid 2-*O*-β-glucoside and l-tryptophan	[109]

### 2.5. Proteomics

Proteins in plants control enzymatic reactions, signal transmission, and intercellular communication. Proteomics focuses on the accurate measurement and examination of protein profiles in the cells, tissues, and organs of living beings. It is especially valuable for pinpointing proteins involved in stress responses and growth regulation. Proteomics can complement genomics by uncovering the functional roles of genes through the proteins they encode [115].

#### 2.5.1. The Technique in Proteomic Analyses

The initial phase of a proteomic investigation involves extracting integral proteins (Figure 5). This process is tailored to the specific organelle or organ under investigation and to the nature of the proteins being analyzed. Mechanical forces are used to break cells and release their contents. Successively, chemical lysis agents, such as Triton X-100, NP-40, Tween 20 and 80, Octyl Glucoside, Octyl Thioglucoside, Big CHAP, Deoxycholate, and Sodium Dodecyl Sulfate, are used to solubilize membrane-bound proteins [116]. In proteomic workflows, the choice of detergent is critical, yet most are incompatible with mass spectrometry and must be removed from samples. Lysis buffers with high critical micelle concentration (CMC) exhibit weak hydrophobic interactions. They can be eliminated via dialysis. Low CMC detergents form micelles more readily and require lower concentrations for effective protein solubilization. They have a small micelle molecular weights, which simplify removal. Selecting the optimal extraction method is no simple task. Standard protocols typically involve precipitation using trichloroacetic acid/acetone, extraction with chloroform/methanol, reduction with dithiothreitol, alkylation using iodoacetamide, and digestion with enzymes such as trypsin and lysyl endopeptidase (Figure 4) [117]. The effectiveness of precipitation depends on the nature of the contaminants, the protein structure, and the analytical goals. Chloroform-methanol is ideal for isolating membrane proteins, while acetone excels at purifying water-soluble proteins. Molecular weight cutoff filters can be used to improve extraction performance. They ensure compatibility between the protein sample and mass spectrometry [118]. Proteomic analyses typically utilize nano-flow liquid chromatography coupled with high-resolution mass spectrometry to achieve comprehensive protein identification and quantification. This equipment can employ distinct acquisition strategies, including data-dependent acquisition (DDA), data-independent acquisition (DIA), and targeted approaches such as parallel reaction monitoring (PRM) or selected reaction monitoring (SRM).

Data-dependent acquisition (DDA), also known as information-dependent acquisition (IDA) or data-directed acquisition, relies on real-time selection of precursor ions directly from MS scans for fragmentation. MS/MS spectra are generated by fragmenting ions that surpass a predefined intensity threshold or meet specific user-defined criteria. While DDA enables broad proteome coverage, it may not fully fragment all detectable peptides, leading to data gaps. Moreover, compared to targeted approaches, DDA typically exhibits lower quantitative accuracy and reproducibility, particularly in complex or low-abundance samples [119].

Data-independent acquisition (DIA) techniques capture fragment ion spectra from all detectable precursors, without the need for prior selection. This approach results in highly complex datasets. Prominent DIA methodologies include collision energy-switching mass spectrometry (MSE), also known as MSAll or all-ion fragmentation (AIF), and sequential window acquisition of all theoretical fragment ion spectra (SWATH) [120].

SWATH-MS streamlines sample preparation. SWATH-MS captures comprehensive fragment-ion maps for all peptide precursor ions using a data-independent acquisition (DIA) strategy without prior selection. The method typically performs 32 MS/MS scans, each spanning 25 m/z isolation windows, enabling high-resolution spectral acquisition across the entire mass range. SWATH-MS allows retrospective interrogation of fragmentation data and leverages spectral libraries to enhance the detection of low-abundance proteins in complex biological samples [121].

An alternative strategy, parallel reaction monitoring (PRM), leverages high-resolution mass spectrometry (HRMS) to detect all fragment ions derived from a selected precursor. PRM facilitates the peptide identification and quantification. Nonetheless, compared to SRM, PRM exhibits slower acquisition rates and lower ion transmission efficiency, which may affect throughput in large-scale studies [122]. Comprehensive protein structure and sequence data are collected in specialized databases. The Universal Protein Resource (UniProt) is a central database for global proteomics, hosting over 220 million annotated protein sequences (2023) [123] and more than 200,000 experimentally resolved 3D structures (2022) [124]. It integrates functional data, domain predictions, and cross-references to 183 external bioinformatics resources [123]. COGs (Clusters of Orthologous Genes), KEGG (Kyoto Encyclopedia of Genes and Genomes), and Pfam organize proteins into evolutionary modules, biochemical pathways, and domain families, respectively. The PRIDE (Proteomics Identification Database; https://www.ebi.ac.uk/pride/, accessed on 13 October 2025) provides proteomics datasets, complete with project metadata, experimental protocols, and quantitative results [124].

#### 2.5.2. Proteomic Insights into Plant Responses to Plastic Exposure

##### MPs and NPs’ Effects on the Protein Levels in Plants

Recent studies have highlighted the diverse impacts of MPs and NPs on protein expression across various species. Ansari et al. reported that MPs from polypropylene, polyethylene, and polyvinyl chloride (100–250 mg L^−1^) reduce protein levels in *Acutodesmus obliquus* [125]. Lian et al. found that polystyrene NPs (diameter 100 nm) impair ion transport proteins [126], whereas Kim et al. observed enhanced protein synthesis in pea seeds exposed to polystyrene MPs (diameter 20 nm) [127]. In rice, Khan et al. showed that polyvinyl chloride MPs (diameter 20 nm) disrupt amino acid metabolism, affecting tryptophan, aspartate, alanine, and glutamate, leading to reduced protein concentrations [128]. Conversely, Shiu et al. demonstrated that MPs increase protein content in the exopolymeric substances of marine phytoplankton [129].

##### MPs and NPs’ Effects on the Cellular Signaling Networks

MPs and NPs have been shown to disrupt protein phosphorylation, a key regulatory mechanism in intercellular signaling and metabolic control. In a study on barley, exposure to fluorescently labeled NPs significantly altered leaf phosphorylation patterns, with downstream effects on nitrogen metabolism, carbon fixation, and other essential metabolic pathways [130].

##### MPs and NPs’ Effects on the Redox Homeostasis

MPs and NPs can modulate the structural dynamics and enzymatic activity of key antioxidant proteins. Wang et al. have demonstrated that smaller NPs induce relaxation of the superoxide dismutase (SOD) backbone, while larger NPs cause structural tightening. Notably, NPs of 100 nm and 500 nm increase the local polarity around tyrosine residues, enhancing SOD’s fluorescence sensitivity [131]. Moreover, Yao et al. have shown that polystyrene NPs (0.1 mg mL^−1^) boost catalase activity by reshaping its conformation and strengthening molecular interactions under acidic conditions [132].

### 2.6. Lipidomics

Lipids are fundamental to cellular function, serving as key components of membrane structure, energy storage, signal transduction, and stress response mechanisms, and mediating interactions in symbiotic and pathogenic relationships. The complete set of lipid species within a cell or organism is referred to as the lipidome. Lipidome analysis, also known as lipidomics, involves the identification, quantification, and functional characterization of lipid molecules [133]. Lipidomics examines the structure, dynamics, and interactions of lipids with proteins, metabolites, and other lipids, offering insights into cellular physiology and metabolic regulation. Figure 5 presents the lipidomic workflow, from sample preparation to data interpretation.

#### 2.6.1. The Technique in Lipidomic Analyses

Plant lipid extraction is commonly performed by liquid–liquid extraction with organic solvents such as hexane, butanol, and petroleum ether under varying conditions. Among the established protocols, the Folch and Bligh-Dyer methods, which utilize chloroform, methanol, and water, are recognized for their efficiency. The Folch method is regarded as the gold standard [134]. Recent research has focused on eco-friendly alternatives to address environmental concerns, including green solvents (such as biodegradable organic compounds), ionic liquids, and supercritical fluids (such as carbon dioxide). Supercritical fluids enable selective extraction, while organic solvents offer advantages in terms of low toxicity, biodegradability, and recyclability. Ionic liquids, defined as non-aqueous salts that remain liquid at room temperature, are a novel class of solvents for lipid recovery. A biphasic system comprising cyclopentyl methyl ether (CPME), methanol, and water has demonstrated comparable efficiency to the Bligh-Dyer method for extracting triacylglycerols from *Lipomyces starkeyi* [135]. However, for total lipid extraction, the Bligh-Dyer method outperformed both 2-methyl tetrahydrofuran (2-MeTHF) and CPME [136]. Mass spectrometry (MS), including LC-MS, mass spectrometry imaging, and ion mobility MS, is the primary technique for lipid detection [137]. Lipidomic analyses require extended chromatographic separation times to resolve structurally diverse lipid species and their isomers effectively. Accelerated methods, while time-efficient, often compromise resolution due to the co-elution of analytes and the ion suppression, which can hinder accurate quantification and identification. Ion mobility spectrometry (IMS) significantly enhances peak capacity by introducing an orthogonal separation dimension. It enables the discrimination of co-eluting compounds based on their collision cross-section (CCS) [138]. The analyzers used in lipidomic analyses include triple quadrupoles (QQQ) for targeted quantification, quadrupole ion traps (QIT) and triple quadrupole-linear ion traps (QTRAP) for enhanced sensitivity and MS/MS capabilities, orbitraps for high-resolution and accurate mass measurements, and quadrupole time-of-flight (Q-ToF) instruments for rapid acquisition with high mass accuracy. Each instrument type offers distinct advantages in terms of resolution, dynamic range, and fragmentation efficiency. Q-ToF and Orbitraps offer high mass accuracy for lipid identification and quantification, but they have low sensitivity. In contrast, QQQ mass spectrometers offer greater sensitivity in multiple reaction monitoring (MRM) modes, making them suitable for analyzing low-abundance lipids. High-resolution mass spectrometers, such as Q-ToF, enable a quantitative analysis of all detectable product ions from a selected precursor ion. The mass spectrometry platforms can employ data-dependent acquisition (DDA) or data-independent acquisition (DIA) methods [135]. High-resolution instruments allow for parallel reaction monitoring. Lipid identification in lipidomics is primarily achieved by matching experimental data of lipid species (fragmentation patterns and mass-to-charge ratios) against those stored in spectral libraries. A range of commercial software platforms support this process, including LipidSearch™ (Thermo Fisher Scientific, Waltham, MA, USA), SimLipid (PREMIER Biosoft; San Francisco, CA, USA), Lipidyzer™ (Sciex; Marlborough, MA, USA), and Lipid Annotator (Agilent Technologies; Santa Clara, CA, USA). Open-access tools, including MS-DIAL (https://systemsomicslab.github.io/compms/msdial/main.html, accessed on 13 October 2025), MS2LDA (https://ms2lda.org/), CFM-ID (https://cfmid.wishartlab.com/), LipidBlast (https://fiehnlab.ucdavis.edu/projects/lipidblast, accessed on 13 October 2025), LIQUID (https://data.pnnl.gov/group/nodes/software/33247, accessed on 13 October 2025), Lipidr (https://www.lipidr.org/), Lipid maps (https://lipidmaps.org/databases, accessed on 13 October 2025), LipidMatch Flow (https://lipidomics.creative-proteomics.com/?utm_source=bing&utm_medium=cpc&utm_campaign=Lipidomics-CPA-35.76-0531&utm_term=lipidomics%20providers&utm_content=0%20Lipidomics-lab%2Fcompany%2Fcro, accessed on 13 October 2025), and LipiDex https://github.com/coongroup/LipiDex (accessed on 13 October 2025), provide versatile solutions for lipidomics analysis.

#### 2.6.2. Lipidomic Studies on Plant Responses to Plastic Derivatives

MPs and NPs can disrupt plant lipid metabolism. Wang et al. demonstrated that polyvinyl chloride MPs stimulate lipid synthesis in root cell membranes while concurrently suppressing the production of lipid-associated metabolites in plants [103]. Exposure to polystyrene MPs (diameter 500 nm) has been shown to enhance sphingolipid metabolism in *Lactuca sativa* [139] and induce membrane lipid peroxidation in *Mirabilis jalapa* L. [140]. Guschina et al. reported that polystyrene MPs (diameter < 70 μm) affect membrane fluidity and integrity, disrupting the lipid bilayer architecture in *Chlorella sorokiniana* [141]. Furthermore, Zhang et al. found that polyethylene NPs (20 and 200 mg kg^−1^) significantly reduce levels of sphingolipids, sterols, glycerophospholipids, and glycolipids in lettuce, while increasing diacylglycerol (DAG) concentrations. Data suggested the activation of the phospholipase C-mediated lipid signaling pathway [142].

### 2.7. Ionomic

Ionomic is an analytical approach used to profile and characterize the ionome, the complete set of mineral nutrients and trace elements (including metals, metalloids, and nonmetals) within an organism. Ionomic studies provide insights into an organism’s functional state and adaptive mechanisms, examining how the ionome changes in response to physiological stimuli, developmental stages, and genetic factors. The ionomic employs elemental analysis platforms (e.g., ICP-MS), bioinformatics pipelines, and genetic tools [143].

#### 2.7.1. The Technique in Ionomic Analyses

Analytical techniques in ionomic research are classified into two categories: those based on atomic electronic properties, including absorption, emission, and fluorescence spectroscopy, and those based on nuclear properties, such as atomic number and radioactivity [144]. Key apparatus include Inductively Coupled Plasma Mass Spectrometry (ICP-MS) and ICP-Atomic Emission Spectrometry (ICP-AES). ICP-MS, compared to traditional atomic absorption spectroscopy, has the advantage of simultaneous quantification of up to 18 elements at parts-per-trillion sensitivity [144]. Other apparata used in ionoc studies include liquid chromatography-photodiode array/mass spectrometry (LC-PDA/MS), capillary electrophoresis-mass spectrometry (CE-MS), X-ray fluorescence (XRF), nuclear magnetic resonance (NMR) spectroscopy, gas chromatography-mass spectrometry (GC-MS), and Fourier transform-ion cyclotron resonance mass spectrometry (FT-ICR/MS) [145]. These apparata offer diverse capabilities for resolving elemental profiles, structural features, and molecular interactions. Bioinformatics tools play a critical role in managing and interpreting the vast datasets generated. The Purdue Ionomics Information Management System (PiiMS) provides an open-access infrastructure for data storage, workflow management, and cross-laboratory analysis, facilitating reproducible and scalable ionomic investigations [146]. Figure 5 illustrates the workflow employed in the ionome analysis methods.

#### 2.7.2. Ionomic Insights into the Effects of Plastic-Derived Compounds on Plants

MPs exert both deleterious and compensatory effects on plant ionomic profiles and growth performance, depending on the context and composition. Tang et al. have demonstrated that polyethylene MPs (diameter 6.5 μm and 150 μm) modulate elemental uptake in *Oryza sativa* seedlings by reducing manganese and arsenic accumulation, and enhancing cadmium and sodium levels [147]. In *Solanum lycopersicum* L., Dainelli et al. have reported that exposure to MPs of polyethylene terephthalate and polyvinyl chloride (diameter 40 and 50 μm) significantly reduces fruit yield, likely due to elevated concentrations of nickel and cadmium in plant tissue [148]. Fu et al. have explored the influence of polyethylene particles (diameter 150–180 µm) of varying molecular weights on *Zea mays.* They demonstrated that polyethylene, polyvinyl chloride, and polypropylene MP (diameter 150 μm and 10 μm) exposure decreases biomass and photosynthetic efficiency, disrupts mineral nutrient metabolism, and alters the rhizosphere elemental composition [149]. Yang et al. have investigated the combined effects of MPs made from polyethylene, polypropylene, and PVC, and of cadmium, on *S. lycopersicum*. Their findings showed that MPs could alleviate Cd-induced toxicity by reducing heavy metal accumulation (Al, Cd, Pb, Cr), limiting uptake of trace (Ni, Mo) and ultra-trace elements (Ag, Co, Sn), and restoring essential nutrient absorption (Ca, Mg, Mn) [150].

## 3. Conclusions

Omics technologies, including genomics, epigenetics, transcriptomics, metabolomics, proteomics, and ionomics, are advancing our scientific comprehension of the biological consequences of plastic pollution in plants, soil, and the soil microbiome. These approaches enable researchers to transcend phenotypic assessments and investigate the intricate molecular and cellular mechanisms activated by MPs and NPs that influence plant physiology. Moreover, they provide detailed insights into how plastic particles influence the composition and function of the soil microbial community, ultimately altering microbial metabolism and the degradation of organic matter. Omics-driven insights lay the groundwork for next-generation sustainable agriculture, enabling the strategic engineering of plants with improved stress resilience and plastic-degrading potential.

Future research should focus on integrating multi-omics approaches to construct a comprehensive understanding of plant and soil microbiome responses to plastic-induced stress. Combining genomics, transcriptomics, metabolomics, and other omics layers, supported by AI-driven data analysis, can enhance the interpretation of complex biological responses and enable predictive modeling of long-term ecological outcomes. To advance environmental risk assessment and mitigation strategies, the scientific community must establish standardized, scalable, and interdisciplinary frameworks. The current lack of harmonized sampling and analytical protocols for plastic derivatives hinders reproducibility, limits cross-study comparability, and obstructs accurate quantification of plastic distribution, transformation, and impact across ecosystems.

## Data Availability

Not applicable.
